# Supplementary material for: Optogenetic control of Protein Kinase C-epsilon activity reveals its intrinsic signaling properties with spatiotemporal resolution
Source: bioRxiv. 2025 Jan 6:2025.01.06.631444. Preprint. [Version 1] doi: 10.1101/2025.01.06.631444 (PMC11741287; doi:10.1101/2025.01.06.631444)
Supplement: Supplement 1 [file NIHPP2025.01.06.631444v1-supplement-1.pdf]

bioRxiv preprint doi: https://doi.org/10.1101/2025.01.06.631444; this version posted January 8, 2025. The copyright holder for this preprint (which was not certified by peer review) is the author/funder, who has granted bioRxiv a license to display the preprint in perpetuity. It is made available under aCC-BY 4.0 International license.

Supplemental Information

Document S1. Figures S1-S6 and Table S4.

Table S1-S3. Excel file containing data too large to fit into a PDF.

Data S1. Excel files and graph analysis files containing data too large to fit into a PDF.
